# Acute kidney injury after coronary artery bypass grafting surgery

**DOI:** 10.1186/cc9521

**Published:** 2011-03-11

**Authors:** P Zeyneloglu, A Pirat, N Veziroglu, A Sezgin, G Arslan

**Affiliations:** 1 Baskent University Hospital, Ankara, Turkey

## Introduction

Acute kidney injury (AKI) after coronary artery bypass grafting (CABG) surgery is associated with increased postoperative morbidity and mortality. The aim of this study was to apply the RIFLE (risk (R), injury (I), failure (F), loss (L) and end-stage kidney disease (E)) criteria in patients after CABG surgery, to identify intraoperative risk factors for occurrence of AKI and to analyze the impact of AKI on mortality.

## Methods

Five hundred consecutive patients who underwent CABG surgery between December 2004 and December 2007 were retrospectively studied. Those who had combined valve and coronary surgery, off-pump surgery and those receiving renal replacement therapy preoperatively were excluded from the study. The primary outcome measure was AKI, defined as ≥50% increase in serum creatinine from baseline.

## Results

The mean age of the patients (74% male) was 60.9 ± 9.8 years. The incidence of AKI was 4%, in which risk occurred in 2%, injury in 1% and failure in 1% of the patients. The cardiopulmonary bypass (CPB) time and duration of the surgery was significantly longer in patients who developed AKI (*P *= 0.024, *P *= 0.002). The amounts of fluid and blood administered and vasopressor requirements during surgeries were similar between patients who developed AKI and those without AKI (*P *> 0.05). The need for intraoperative cardiopulmonary resuscitation (CPR), the use of intra-aortic balloon pump (IABP) and total circulatory arrest (TCA) was significantly higher in AKI patients (*P *= 0.002, *P *= 0.001 and *P *= 0.036, respectively). When compared with non-AKI patients, postoperative mortality for patients experiencing AKI was significantly high (*P *= 0.001). There was a significant positive correlation between presence of postoperative mortality and AKI (*r *= 0.232, *P *< 0.001).

## Conclusions

The results suggest that AKI develops in 4% of patients after CABG surgery. Intraoperative risk factors for occurrence of AKI include longer duration of surgery, CPB time and requirements of CPR, IABP and TCA usage. In addition, postoperative development of AKI is associated with mortality.
